# Standards of conduct and reporting in evidence syntheses that could inform environmental policy and management decisions

**DOI:** 10.1186/s13750-022-00269-9

**Published:** 2022-04-19

**Authors:** Andrew S. Pullin, Samantha H. Cheng, Josephine D’Urban Jackson, Jacqualyn Eales, Ida Envall, Salamatu J. Fada, Geoff K. Frampton, Meagan Harper, Andrew N. Kadykalo, Christian Kohl, Ko Konno, Barbara Livoreil, Dakis-Yaoba Ouédraogo, Bethan C. O’Leary, George Pullin, Nicola Randall, Rebecca Rees, Adrienne Smith, Romain Sordello, Eleanor J. Sterling, Will M. Twardek, Paul Woodcock

**Affiliations:** 1 Collaboration for Environmental Evidence, Conwy, UK; 2 Center for Biodiversity and Conservation, American Museum of Natural History, New York, USA; 3 Natural Resources Wales, Cambria House, 29 Newport Rd, Cardiff, CF24 0TP UK; 4 European Centre for Environment and Human Health, College of Medicine and Health, University of Exeter, Knowledge Spa, Truro, TR1 3HD UK; 5 The Swedish Research Council for Environment, Agricultural Sciences and Spatial Planning, Formas, Stockholm, Sweden; 6 Bangor University, Bangor, UK; 7 University of Jos, Jos, Nigeria; 8 Southampton Health Technology Assessments Centre, Faculty of Medicine, University of Southampton, Southampton, UK; 9 Canadian Centre for Evidence-Based Conservation, Department of Biology and Institute of Environmental and Interdisciplinary Science, Carleton University, Ottawa, Canada; 10 Julius Kühn Institute (JKI), Federal Research Centre for Cultivated Plants, Institute for Biosafety in Plant Biotechnology (SB), Quedlinburg, Germany; 11 School of Natural Sciences, Bangor University, Bangor, UK; 12 30 rue Lamartine, 83340 Le Luc, France; 13 Direction de L’Expertise, Muséum National d’Histoire Naturelle (MNHN), 75005 Paris, France; 14 Centre for Ecology & Conservation, College of Life and Environmental Sciences, University of Exeter, Penryn Campus, Penryn, UK; 15 Department of Environment and Geography, University of York, York, UK; 16 University of York, York, UK; 17 Centre for Evidence Based Agriculture, Harper Adams University, Newport, UK; 18 EPPI-Centre, UCL Social Research Institute, University College London, London, UK; 19 Canadian Centre for Evidence-Based Conservation, Carleton University, Ottawa, Canada; 20 UMS PatriNat OFB-CNRS-MNHN, Paris, France; 21 Center for Biodiversity and Conservation, American Museum of Natural History, New York, NY 10024 USA; 22 Fish Ecology and Conservation Physiology Lab, Carleton University, 1125 Colonel By Dr, Ottawa, ON K1S 5B6 Canada; 23 Joint Nature Conservation Committee, Monkstone House, Peterborough, PE1 1JY UK

**Keywords:** CEEDER, CEESAT, Evidence synthesis, Evidence-informed decision making, Review reliability, Environmental evidence

## Abstract

Accurate, unbiased and concise synthesis of available evidence following clear methodology and transparent reporting is necessary to support effective environmental policy and management decisions. Without this, less reliable and/or less objective reviews of evidence could inform decision making, leading to ineffective, resource wasteful interventions with potential for unintended consequences. We evaluated the reliability of over 1000 evidence syntheses (reviews and overviews) published between 2018 and 2020 that provide evidence on the impacts of human activities or effectiveness of interventions relevant to environmental management. The syntheses are drawn from the Collaboration for Environmental Evidence Database of Evidence Reviews (CEEDER), an online, freely available evidence service for evidence users that assesses the reliability of evidence syntheses using a series of published criteria. We found that the majority of syntheses have problems with transparency, replicability and potential for bias. Overall, our results suggest that most recently published evidence syntheses are of low reliability to inform decision making. Reviews that followed guidance and reporting standards for evidence synthesis had improved assessment ratings, but there remains substantial variation in the standard of reviews amongst even these. Furthermore, the term ‘systematic review’, which implies conformity with a methodological standard, was frequently misused. A major objective of the CEEDER project is to improve the reliability of the global body of environmental evidence reviews. To this end we outline freely available online resources to help improve review conduct and reporting. We call on authors, editors and peer reviewers to use these resources to ensure more reliable syntheses in the future.

**Supplementary Information:**

The online version contains supplementary material available at 10.1186/s13750-022-00269-9.

## Background

The number of primary research articles that potentially provide evidence to inform environmental management is increasing year on year. Identifying and making sense of the nature and findings of relevant articles when making decisions is an almost impossible task for most decision makers [6, 12]. Consequently, evidence syntheses, usually in the form of reviews, that collate and synthesise findings from primary research, are a key step to enabling evidence-informed decision making [9]. Unfortunately, as with primary research, syntheses can be misleading if they are susceptible to bias or if insufficient reporting of methods hides limitations that impact on the reliability of findings [4]. Biased findings could misinform decision makers, with potentially significant consequences for the environment and society.

To address problems of bias and reliability, systematic methods for producing rigorous evidence syntheses to inform policy and practice have become increasingly standardised across various fields over the last 30 or so years [3]. Such methods are applied both to the production of syntheses aggregating research findings (Systematic Reviews) and to the creation of descriptive maps that collate and configure (arrange in a logical way) existing research (Systematic Maps). For environmental management, the first guidance on how best to conduct Systematic Reviews was published in 2006 [11] and subsequently, the Collaboration for Environmental Evidence (CEE) has provided guidance on evidence syntheses [2]. This methodological guidance, available to both authors and editors, sets out conduct and reporting standards that can reduce bias and increase reliability, but it is not clear to what extent such guidance is followed in evidence syntheses currently being published.

Decision makers may be unaware of the potential for bias in evidence syntheses, or may not have the appropriate skills or time to critically evaluate the reliability of findings for their evidence needs. This uncertainty in the provision of scientific evidence to inform management and policy decisions has gained little attention to date in environmental evidence synthesis. O’Leary et al. [8] investigated evidence synthesis reliability in environmental research using a synthesis appraisal tool (CEE Synthesis Appraisal Tool: CEESAT) developed initially by Woodcock et al. [14], that focussed on key aspects of review conduct and reporting. Using a sample of 92 reviews published in 2015 they found very low reliability ratings for most.

The CEEDER (CEE Database of Evidence Reviews) project was initiated in 2018 to provide a database of available evidence syntheses specifically on impacts of human activities or effectiveness of interventions relevant to issues in the environmental field [7]. Through extensive searching of both commercially published and grey literature sources, and screening using standardised eligibility criteria, a comprehensive global database of evidence syntheses was compiled and is updated on a regular basis [7]. CEEDER provides an opportunity to assess the reliability of evidence syntheses as they are published.

In this paper we use the generic term ‘evidence synthesis’ to mean the process of combining scientific information from multiple studies that have investigated the same question, to come to an overall understanding of what they found. As such, evidence syntheses are published or otherwise made available under many different names. Currently CEEDER recognises two forms of evidence synthesis:

1. Evidence reviews (including literature reviews, critical reviews, Systematic Reviews) are defined here as syntheses of primary studies (narratively and/or quantitatively) that address a question of cause and effect and provide (or claim to provide) an aggregate measure or estimate of effect (e.g., impact of anthropogenic activity or effectiveness of an intervention).
2. Evidence overviews (including Systematic Maps, scoping reviews, Evidence Gap Maps) are similar but usually address a broader question (often involving multiple causes and effects) and collate and configure evidence but do not provide an aggregate measure or estimate of effect.

More details can be found in Konno et al. [7] and on the CEEDER website (https://environmentalevidence.org/ceeder/).

### The subject coverage of evidence syntheses in CEEDER

CEEDER uses specific eligibility criteria to identify evidence syntheses within its subject scope (see https://environmentalevidence.org/ceeder/about-ceeder/). This means that some subjects at the margins of environmental science and management are currently excluded, for example, some aspects of public health, toxicology, and plant and animal science. Nevertheless, CEEDER includes a wide range of environmental science subjects and can reveal which of these have been a particular focus of attention over a period of time, and which have been somewhat neglected. An example of a popular overarching subject of syntheses currently in the CEEDER database (articles published between 2018 and 2020) is global change, in the context of both impacts and interventions (e.g., mitigation efforts). Not surprisingly, there appears to be a particular interest in the impacts of climate change (e.g., changes in temperature, precipitation) on ecosystems, crops, greenhouse gas emissions, and soil organic carbon stocks. In terms of mitigation some agricultural practices (e.g., tillage options) are common. Other popular subject elements include: biochar use in soils, pollutant removal through wastewater treatment, greening urban environments, and habitat restoration.

Examples of subjects currently not found or rarely found in the database include: impacts of blue/green hydrogen technology (0 syntheses), zoonotics (2 syntheses), blue carbon sequestration (two syntheses compared to 44 syntheses involving soil organic carbon), genetically modified organisms (4 syntheses), permafrost (2 syntheses), glacier melt (1 synthesis), poaching (1 synthesis) and wildlife trade (0 syntheses).

As an example of change in frequency of a subject over time, “ecosystem services” increased in frequency year by year in (author defined) keywords: 2018: 11 syntheses, 2019: 15 syntheses, 2020: 18 syntheses.

### The geographic origin of evidence syntheses by author affiliation

**Fig. 1:**
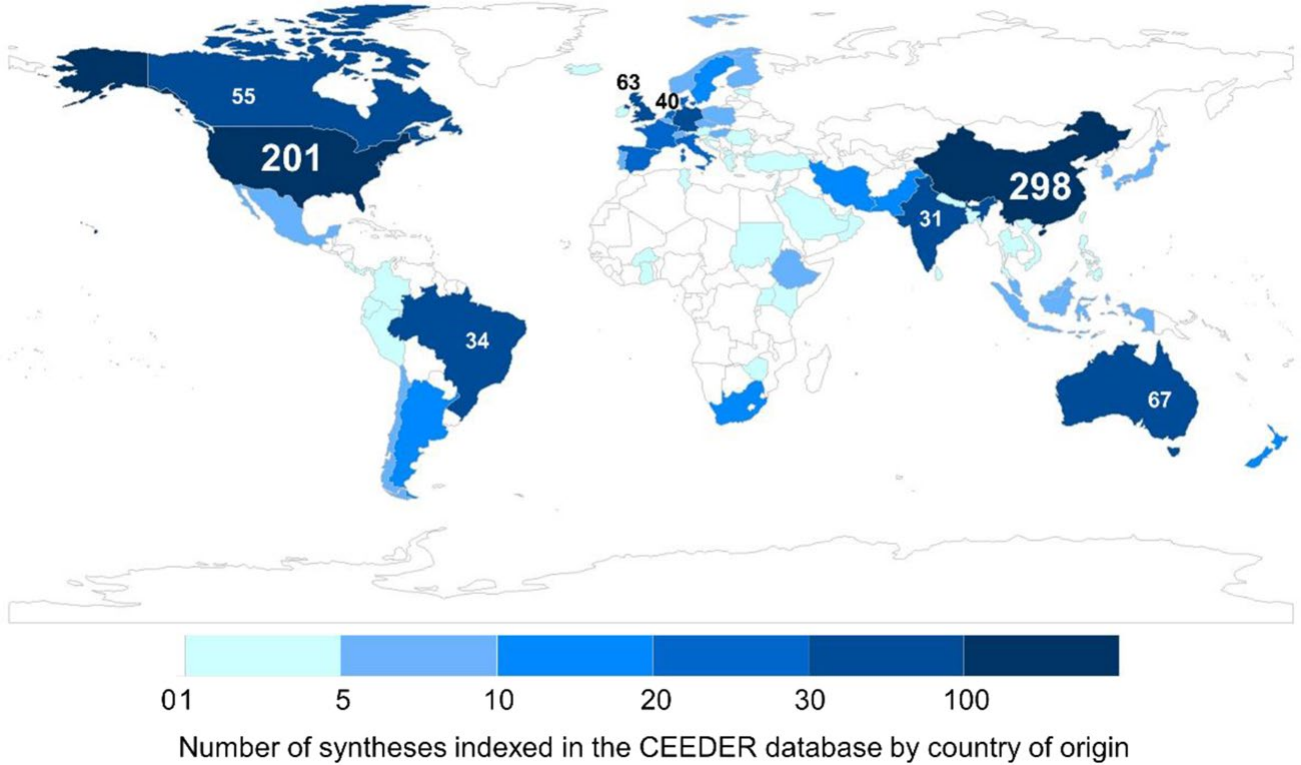
Number of evidence syntheses indexed in the CEEDER database by country of origin (corresponding authors’ affiliation). Some syntheses are counted multiple times as authors may be affiliated with multiple institutions located in different countries. Frequencies are provided for those that exceed 30 (China, USA, Australia, UK, Canada, Germany, Brazil and India)

Based on corresponding authors’ affiliation to countries, evidence syntheses included within CEEDER currently represent 71 different countries (Fig. 1). Of these, eight countries dominate, producing more than 30 evidence syntheses each and collectively accounting for 67.2% of the total number of evidence syntheses within CEEDER (Fig. 1; numbers for each country can be found in Additional File 1).

## Objectives

The CEEDER database now contains over 1000 syntheses published in the 3 years (2018–2020). We report our findings on their conduct and reporting and present an overall assessment of their reliability. We assess to what extent the provision of Systematic Review guidance can improve conduct and reporting and thus more reliable syntheses. We also explore whether syntheses that claim to be Systematic Reviews are as reliable as we would expect if guidance and standards available for their conduct are followed (e.g., [2]). Finally, since a major objective of the CEEDER project is to improve the reliability of the global body of environmental evidence syntheses, we describe some open access resources available to authors, editors and peer reviewers to improve the replicability and reliability of future environmental evidence syntheses.

At present CEEDER contains only English language articles and excludes many evidence syntheses conducted on other aspects of environmental management (CEEDER may be expanded in scope to include more of these in the future).

## Methods

### Development and management of the CEEDER database

The rationale for the CEEDER project and the methods used to compile the CEEDER database have been described in detail elsewhere [7]. In brief, CEEDER Editors and Review College members (there are 45 members at the time of writing) critically assess each synthesis article (including Additional File 1) for its reliability in terms of replicability of conduct and transparency of reporting using an updated version of the original CEESAT tool (https://environmentalevidence.org/ceeder/about-ceesat/). (See Table 1 for glossary of terms, please note that conduct can only be assessed based on what is reported).

**Table 1:** Glossary of terms describing key characteristics of evidence synthesis conduct and reporting

Reliability	The extent to which an evidence synthesis can be trusted as an estimate of the truth
Replicability	The extent to which the conduct of an evidence synthesis is reported so that it could be replicated by a third party
Transparency	The extent to which the evidence synthesis methods, analyses, data, and limitations are reported openly
Potential for bias	The likelihood that the conduct of an evidence synthesis might provide misleading results or findings

The CEESAT tool has two versions; one for evidence reviews with 16 criteria and one for evidence overviews with 11 criteria, which cover the main stages that would be expected to be clearly reported in a robust evidence review (i.e., a Systematic Review) or a robust evidence overview (i.e., a Systematic Map). These stages include planning, searching, screening, critical appraisal of included studies, data extraction and coding, data synthesis, and limitations. Each criterion is rated by assigning one of four categories:

- Gold—The highest standards of conduct and reporting that can reasonably be expected for high replicability and low potential for bias.
- Green—Standards of conduct and reporting that enable replication and reduce potential bias.
- Amber—Standards of conduct and reporting that lack some key elements that enable replication and reduce potential for bias.
- Red—Standards of conduct and reporting that lack most key elements that enable replication and reduce potential for bias.

Note that for the purposes of descriptive analyses, these nominal classes can be regarded as a four-point ordinal scale, with gold representing the highest standard and red representing the lowest standard.

Each article is assessed by a minimum of two reviewers from the Review College and, after any differences have been checked and resolved by an editor (and the original reviewers if necessary), the article is assigned one of the gold, green, amber, or red categories. CEESAT has been developed to provide a checklist that is as objective and repeatable as possible but considerable subjectivity remains and this is a major reason for requiring a minimum of two reviewers per article and editorial checks.

### Analysis of evidence syntheses in the CEEDER database

We used the CEEDER database to extract CEESAT metadata from the entire database of 1058 evidence syntheses published between 2018 and 2020.

#### Reliability of evidence reviews and overviews

CEESAT was used to appraise the two types of synthesis currently included in CEEDER, ‘Evidence reviews’ (n = 924) and ‘Evidence overviews’ (n = 134), using different versions of the tool to address different expectations of their methodology. For our analysis, the CEESAT ratings were collated across all evidence reviews and overviews, using the appropriate CEESAT versions, to provide an overall estimate of reliability for each. It is important to note that the CEESAT ratings take into account both the conduct and reporting of synthesis methodology and the limitations imposed on analysis by the state of the primary data. For an example of the latter, statistical meta-analysis may not be possible due to limitations imposed by lack of available data (CEESAT criteria 7.1 to 7.3). Note also that critical appraisal (CEESAT criteria 5.1 to 5.2) and appropriateness and replicability of the synthesis method (CEESAT criteria 7.1 to 7.3) do not apply to evidence overviews.

#### Does citing/using methodological guidance or reporting checklists improve reliability of evidence reviews?

We compared the modal assessment ratings (gold, green, amber or red) for each CEESAT assessment item between subsets of evidence reviews only (n = 924) to look at whether evidence reviews that used available guidance for Systematic Reviews differed in their CEESAT ratings compared to those which did not cite any guidance documents. Considering the ordinal nature of the assessments, we use difference between the modal assessment ratings (represented by the colours) to represent differences in standards of methodological conduct and reporting between subsets of evidence reviews.

Three subsets of evidence reviews were compared:

- Evidence reviews citing any version of the CEE Guidelines and Standards for Evidence Synthesis in Environmental Management (e.g., [2]).
- Evidence reviews citing the Preferred Reporting Items for Systematic Reviews and Meta-Analyses (PRISMA, http://www.prisma-statement.org/), including any extension. PRISMA is often cited as being followed even though it is a reporting checklist rather than providing guidance on conduct.
- Evidence reviews citing no guidance.

#### Does reliability of evidence reviews improve when authors claim to have conducted a systematic review?

We searched each evidence review to see if authors claimed to have conducted a Systematic Review but did not register their review with CEE. We only accepted clear statements to this effect (e.g., ‘we conducted a systematic review’) in any part of the article and did not include those that claimed to have conducted a ‘systematic literature search’ or a ‘systematic literature review’ as these potentially refer to the search only. We then compared the CEESAT ratings of this subset against those of all evidence reviews. This subset is bigger than, but includes, those that cite the CEE Guidelines and Standards mentioned above.

## Results

### Reliability and replicability of evidence reviews and overviews by CEESAT criteria

**Fig. 2:**
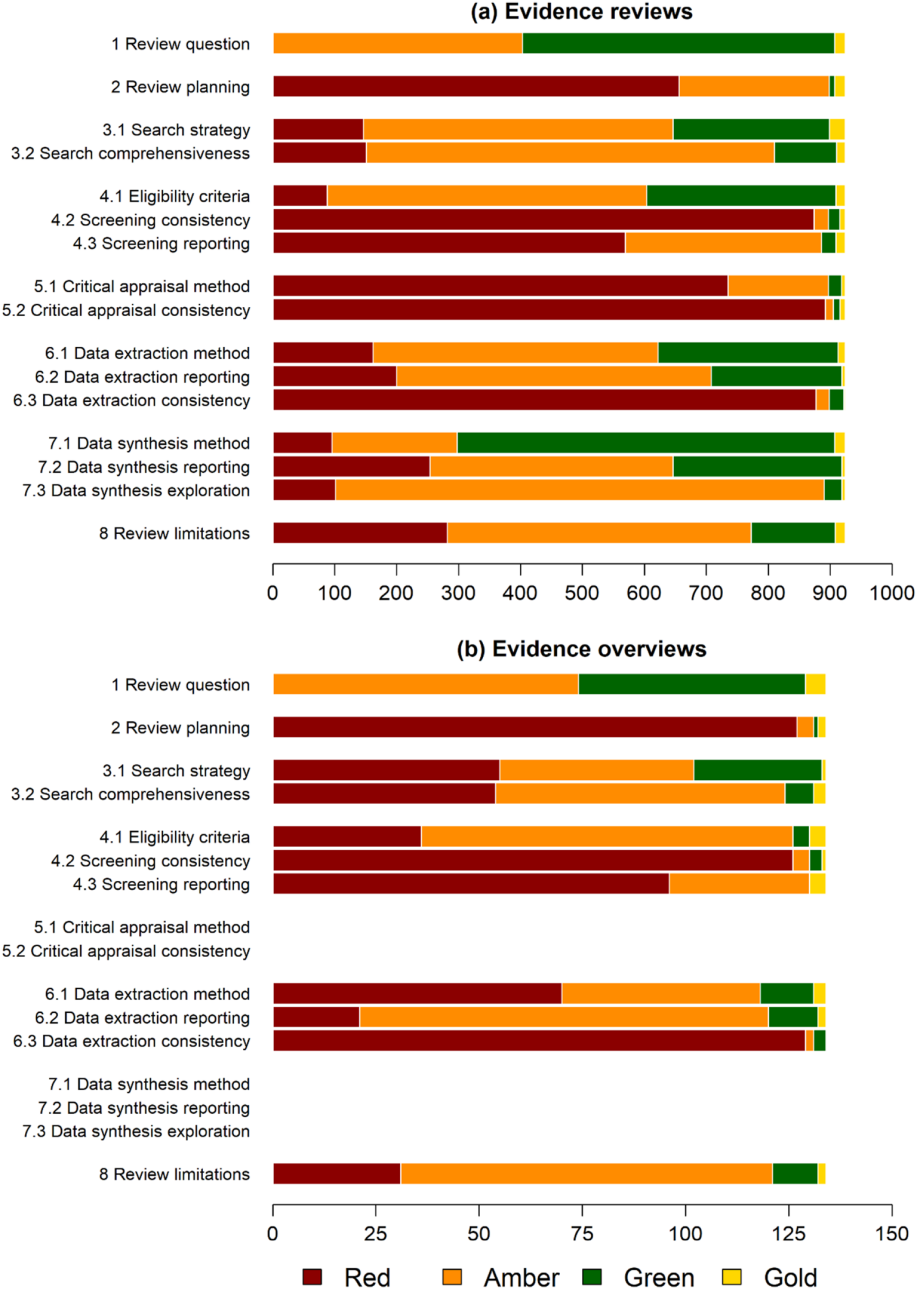
The distribution of CEESAT ratings for each criterion for evidence reviews (n = 924, top) and evidence overviews (n = 134, bottom) published between 2018 and 2020. Note, no red category is included for Criterion 1 as this is an eligibility criterion for inclusion in the CEEDER database (red articles for criterion 1 are excluded from CEEDER). CEESAT criteria 5 and 7 are not applied to overviews

The distribution of assessment ratings (gold, green, amber, red) for each CEESAT criterion among all evidence reviews is shown in Fig. 2a. Overall, “red” and “amber” assessments dominate over “green” and “gold”, suggesting the majority of reviews have problems with transparency, replicability, and potential for bias. Particular problem areas appear to be no formal review planning (protocols) and poor reporting of methods (criterion 2), no formal critical appraisal of primary studies (criterion 5), poor reporting of screening decisions and outcomes (criterion 4.3) and lack of consistency checking of reviewer decisions at screening, critical appraisal, and data extraction stages (criteria 4.2, 5.2 & 6.3). Excepting criterion 1 (clear review question), where reds are excluded before assessment, reds and ambers constitute more than 50% of assessments in all but criterion 7.1 (appropriateness and replicability of the synthesis/meta-analysis method). The assessments for overviews (Fig. 2b) show a similar pattern, highlighting the same problems of planning, reporting, and consistency of decisions.

### Does citing/using methodological guidance or reporting checklists improve reliability of evidence reviews?

**Fig. 3:**
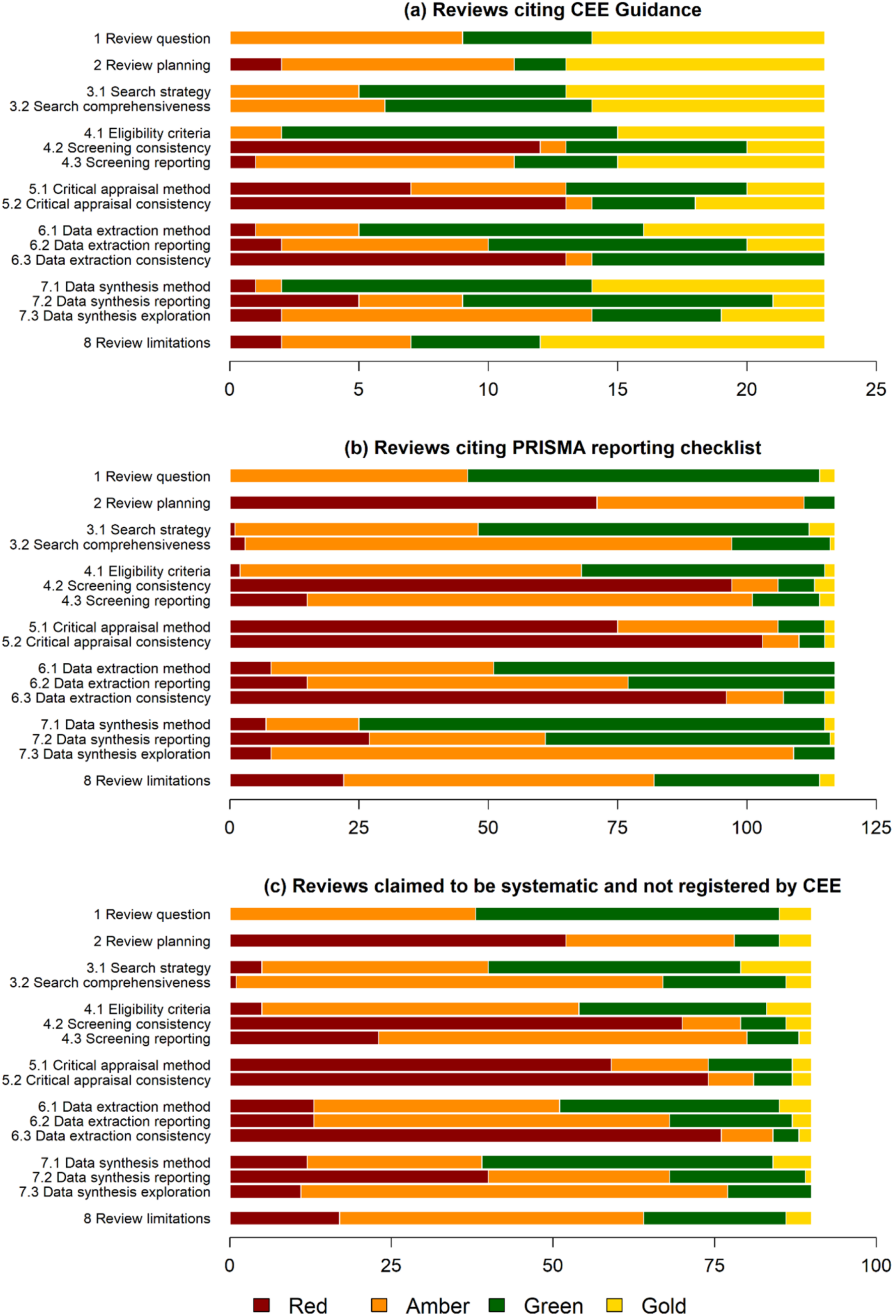
Distribution of assessment ratings in **a** evidence reviews citing CEE Guidelines (including CEE Systematic Reviews) n = 23/924; **b** evidence reviews citing PRISMA reporting checklist n = 117/924; and **c** evidence reviews where authors claim to have conducted a Systematic Review but were not registered by CEE n = 90/924. X axis shows absolute numbers of evidence reviews

Authors cited the use of CEE Guidelines in 23 of 924 evidence reviews (including eight that were registered with CEE). The distribution of assessment ratings is shown in Fig. 3a. The comparison of modal assessment ratings between all evidence reviews, those not citing any guidance, and those that cite CEE Guidelines (Table 2) shows a notable increase in ratings in the latter. On examination, this is caused predominantly by the subset of reviews that followed the full CEE process, although this is a relatively small number (eight reviews; data not shown separately in Table 2). For example, for some criteria (e.g., 5.1 critical appraisal methods) there is a clear bimodal distribution of assessments caused mainly, but not exclusively by the difference between those eight which followed the full CEE process through registration (mostly contributing to higher ratings) and those which only cited the guidance (mostly contributing to lower ratings). Even so, the latter group shows improvement in the majority of criteria.

**Table 2:**
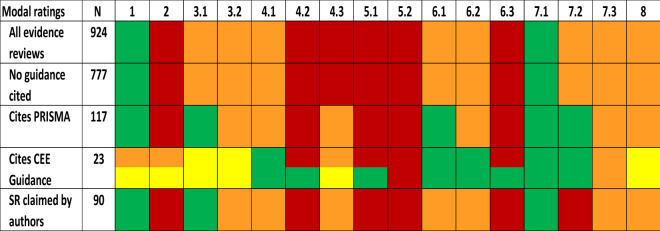
Comparison of modal assessment ratings (colours) for all evidence reviews and selected subgroups

When two colours are given this is due to bimodal pattern in assessments. CEESAT criteria on top line are: 1. Review question, 2. Review planning, 3.1 Search strategy, 3.2 Search comprehensiveness, 4.1 Eligibility criteria, 4.2 Screening consistency, 4.3 screening reporting, 5.1 Critical appraisal method, 5.2 Critical appraisal consistency, 6.1 data extraction method, 6.2 Data extraction reporting, 6.3 Data extraction consistency, 7.1 Data synthesis method, 7.2 Data synthesis reporting, 7.3 Data synthesis exploration of variability, 8 Review limitations. SR stands for Systematic Review

The PRISMA reporting checklist was more frequently used with authors citing its use in 117/924 evidence reviews. The distribution of assessment ratings is shown in Fig. 3b. The comparison of modal assessment ratings between all evidence reviews and the subset that cite PRISMA reporting guidance is shown in Table 2: a marginal increase in some CEESAT ratings can be seen by those reviews that cite PRISMA.

### Does reliability of evidence reviews improve when authors claim to have conducted a systematic review?

Of the evidence reviews, 90/924 claimed to be Systematic Reviews but were not registered with CEE. The distribution of assessment ratings for reviews claiming to be Systematic Reviews (Fig. 3c and Table 2) is not radically different from those of all evidence reviews (Fig. 2, Table 2); almost all evidence reviews fail to meet the criteria that commonly define Systematic Reviews.

## Discussion

Our results demonstrate that a majority of recently published evidence syntheses on impacts of human activities on the environment or effectiveness of interventions for environmental management are of low reliability to inform decision making. In particular, the evidence syntheses lack rigour and transparency and would therefore be expected to lack replicability at both the planning and conduct stages. Specific problem areas of the evidence syntheses are: lack of an *a-priori* protocol or even a replicable method section in the paper itself; inadequate reporting of screening decisions on what primary research is included or excluded and why; and a lack of checking for consistency of decisions among reviewers at key screening, critical appraisal, and data extraction stages. Importantly, as shown in Table 2, evidence syntheses, including those that cited CEE Guidelines, often lacked any critical appraisal of their included studies (CEESAT criteria 5.1 and 5.2). In such cases, the findings of all the included studies in the review are essentially treated as having equal validity for the synthesis despite the risk of bias differing according to primary study design [1]. This is evident even in many meta-analyses in CEEDER, which may be weighted to take account of sample size (using inverse of variance) but do not appraise and weight studies for risk of bias.

General shortcomings in the reliability of evidence syntheses have been known for more than 30 years and in environmental management research for at least 20 [10], with more recent research continuing to reveal concerns [8, 11, 14, 15]. Guidance on conducting and reporting more rigorous evidence syntheses has been available during this time, both from other disciplines (Cochrane, Campbell, PRISMA) and from within the environmental management community [2]. Our results suggest that available guidance improves review reliability when followed (but not necessarily when cited). However, only approximately 1% of the sample followed the CEE Guidelines and process in full whilst another 1% cited the guidelines only, and so this had had little impact on the conduct of evidence syntheses as a whole. Comparing our CEESAT ratings from 2018 to 2020 with those of evidence syntheses published in 2015 [8] does not provide a clear signal that this situation is getting any better over time. Quantitative comparison with 2015 data is not possible as, even though the current version of CEESAT has been developed directly from the original version of Woodcock et al. [14] and the basic criteria of assessment (searching, screening etc.,) have not changed, CEESAT has developed over this time in terms of definitions of some standards.

Another concern is the apparent misuse of the term ‘Systematic Review’ when authors of evidence syntheses claim to have conducted a Systematic Review without meeting the expected standards. Systematic Review is a methodology that has procedures and standards recognised by international bodies, but the term was used by approximately 10% of our sample with no apparent intent in their methods section to meet these standards. Our analysis shows that these claimed Systematic Reviews were only marginally better than all reviews on some reliability criteria and none met the recognised Systematic Review standards. The unsubstantiated claim to have used a recognised scientific methodology is a serious issue and undermines progress in both evidence synthesis and evidence-informed policy. We call on all journal editors and peer reviewers to challenge claims by authors to have conducted a Systematic Review by using recognised standards and checklists in the peer review process (see next section).

The objective of the CEESAT assessments of evidence syntheses and the methodology it employs is to provide an estimate of how reliable an evidence synthesis could be if used to inform decision-making. The assessments do not tell us whether the findings of a particular review are a good or poor estimate of the truth (although the absence of any critical appraisal assessment in an evidence synthesis should raise concerns that results of the included studies, and hence also of the evidence synthesis, could be biased). CEESAT criteria essentially provide a risk assessment of the use of review findings. The criteria do not take into account the possibility of malpractice or errors in analysis [8, 14]. The frequency of these problems is unknown in environmental evidence syntheses as far as we are aware, although errors undoubtedly occur. A further consideration is that the reliability of many evidence syntheses is limited by the reliability of the eligible primary studies. Limited numbers of studies and studies of low validity can limit data synthesis (CEESAT criterion 7) despite high standards of conduct and reporting.

CEEDER’s scope excludes many evidence syntheses that do not seek to measure effectiveness or impact. Although not tested in our analysis, it seems plausible that these other evidence syntheses would likely exhibit similar limitations to those which are captured in CEEDER. It is possible that authors of eligible evidence syntheses may not have considered them in the context of providing evidence to inform policy or practice. However, we do not view this as a justification for low replicability or high potential for bias. It could also be claimed that the extra effort required to meet CEESAT criteria would not change the review findings and would therefore be wasted. Whilst this might be the case in some instances, the value of transparency and replicability in the context of providing trusted sources of evidence still stands in our view. Furthermore, separating those reviews for which the results would change from those that it would not, prior to or after review conduct, would be impossible. Improving standards of scientific practice should therefore be universal.

An understanding of why evidence reviews are of low reliability would be helpful for improving future evidence synthesis practice. Possible reasons for poor reliability could include lack of time or funding, lack of methodological awareness, disagreement over the need for some criteria, or meeting high standards of conduct being regarded as disproportionate to the impact of the evidence or risk of bias. However, the task of raising some, if not all, standards is being made increasingly easier by a suite of new resources freely available to authors, and we present some examples below. Now that both CEEDER and other resources are available to authors and editors, it will be interesting to monitor to what extent they are used and what impact this will have on evidence provision and use.

### Increasing reliability of evidence syntheses

Whilst the primary objective of the CEEDER project is to provide a service to evidence users, a secondary objective is to increase the general reliability of evidence syntheses (which in turn improves the service). Both CEE and its CEEDER project provide access to a suite of free resources to help authors, editors and peer reviewers improve conduct and reporting (Table 3). The CEE Guidelines and Standards for evidence synthesis can be accessed and used by all authors regardless of whether they want to follow the full CEE process or not. The CEE Guidelines and Standards are periodically updated (e.g., improved guidance on how to plan and conduct critical appraisal is expected to be published during 2022). Free online training in the conduct of Systematic Reviews and Maps is also available from the CEE website. The most up to date CEESAT criteria (for both evidence reviews and overviews) are freely accessible from the CEEDER webpages and can be used by authors to plan and conduct evidence syntheses, and by editors and peer reviewers as a checklist of standards. To improve reporting we encourage the use of the ROSES reporting checklist which is specifically for environmental evidence syntheses [5]. The PRISMA reporting checklist can also be used but neither of these should be cited as providing guidance on review conduct. The Julius Kühn-Institut (JKI) and CEE jointly provide the free online tool CADIMA for authors to manage the review process from searching through to critical appraisal and data extraction. Similar software is also available from other sources and much of it is free to use.

**Table 3:** Summary of free resources for authors, editors and peer reviewers from CEE and partners

Resource	Link	Use	Comments
CEE Guidelines and Standards	https://environmentalevidence.org/information-for-authors/	Open access guidance for the conduct of systematic reviews and systematic maps	Can be used to improve reliability of any evidence synthesis
CEE Online Training	https://environmentalevidence.org/training-workshops/	Free resources to provide initial training in evidence synthesis	More specific training events are available
CEEDER checklists	https://environmentalevidence.org/ceeder/	Criteria and checklist for standards of conduct and reporting	Can be used by authors, editors and peer reviewers. Based on the most up to date version of CEESAT
ROSES	https://environmentalevidence.org/roses/	Reporting standards checklist for systematic reviews and maps	Can be used by authors, editors and peer reviewers
CADIMA	https://www.cadima.info/	A free web tool facilitating the conduct and reporting of systematic reviews and maps and other reviews	Can be used by authors of evidence reviews/overviews
PROCEED	www.proceedevidence.info	Title registration and protocol submission system for all environmental reviews	Can be used by authors of evidence reviews/overviews
CEE Critical Appraisal Tool	https://environmentalevidence.org/cee-critical-appraisal-tool/	A tool to facilitate study validity assessment of primary studies	Can be used by authors of evidence reviews/overviews

To address improvement in more specific stages of evidence synthesis, CEE is establishing two new resources. To raise the standard of planning and replicability of evidence syntheses CEE, in partnership with JKI, has launched a website for free registration of review titles and protocols (PROCEED). This will enable authors to have their protocols published in advance of conducting the review (protocols will be given a Digital Object Identifier [DOI] and be citable). To address the standard of critical appraisal of primary studies CEE has developed a prototype critical appraisal tool that provides a stepwise process for identifying and appraising risk of bias in primary studies (Table 3).

## Conclusions

One of the aspirations of the environmental science community is to provide scientific evidence that can inform decisions for better environmental management. Publication of large numbers of evidence syntheses of low reliability and high potential for bias is likely to hinder progress in this direction. The scientific community has a responsibility to serve the evidence user community better by providing more trustworthy evidence syntheses. The CEEDER project aims not only to report on the state of evidence reviews and overviews but also to provide free access to support for authors, journal editors and peer reviewers to produce more reliable evidence syntheses by increasing transparency, improving reporting and reducing of the potential for bias.

The CEEDER database provides a lens through which we can assess the state of the environmental evidence base. The current picture it provides is one of low reliability of evidence synthesis caused by poor conduct and reporting. Much of this may be due to lack of awareness in the environmental science community of the development of synthesis methodology. Fortunately, the adoption of methodologies and raising standards can be addressed, reasonably easily in many cases. We call on authors, editors and peer reviewers to use the available resources (e.g., Table 3) to ensure more reliable reviews so that the research community provides a better evidence service to inform environmental policy and management and reduce waste of scarce resources.

## Supplementary Information

**Additional file 1.** Table of countries showing frequency of contribution to evidence reviews and overviews using corresponding authors’ affiliation.

## Data Availability

CEEDER is an open access database and can be accessed at https://environmentalevidence.org/ceeder/.
