# Supplementary material for: Standards of conduct and reporting in evidence syntheses that could inform environmental policy and management decisions
Source: Environ Evid. 2022 Apr 19;11:16. doi: 10.1186/s13750-022-00269-9 (PMC11378768; doi:10.1186/s13750-022-00269-9)
Supplement: Supplementary file 1 — Additional file 1. Table of countries showing frequency of contribution to evidence reviews and overviews using corresponding authors’ affiliation. [file 13750_2022_269_MOESM1_ESM.docx]

Pullin et al. 2022. Standards of conduct and reporting in evidence syntheses that could inform environmental policy and management decisions.

**Additional File 1**

Table of countries showing frequency of contribution to evidence reviews and overviews using corresponding authors’ affiliation. To examine geographic foci of review activity, the country of each corresponding author’s affiliation was recorded for each of the 1058 reviews/overviews. Country names were coded according to International Organization for Standardization (ISO)’s 3166 Alpha-3 Country Code (https://www.iso.org/iso-3166-country-codes.html, accessed on 26 June 2021; e.g. United States of America is coded as USA). If multiple countries apply to one corresponding author, or there were multiple corresponding authors, all affiliations were recorded. If there was no indication of correspondence, the first author affiliation was used.

| Country | Frequency |
| --- | --- |
|  |  |
| China | 298 |
| USA | 201 |
| Australia | 67 |
| UK | 63 |
| Canada | 55 |
| Germany | 40 |
| Brazil | 34 |
| India | 31 |
| France | 29 |
| Italy | 28 |
| Denmark | 22 |
| The Netherlands | 21 |
| Spain | 20 |
| Sweden | 19 |
| South Africa | 13 |
| Iran | 11 |
| New Zealand | 11 |
| Argentina | 10 |
| Pakistan | 10 |
| Ethiopia | 9 |
| Finland | 9 |
| South Korea | 9 |
| Mexico | 9 |
| Poland | 9 |
| Belgium | 8 |
| Switzerland | 8 |
| Chile | 8 |
| Portugal | 8 |
| Hungary | 7 |
| Japan | 7 |
| Czech Republic | 5 |
| Indonesia | 5 |
| Malaysia | 5 |
| Norway | 5 |
| Singapore | 5 |
| Austria | 4 |
| Kenya | 4 |
| Oman | 4 |
| Estonia | 3 |
| Ghana | 3 |
| Greece | 3 |
| Ireland | 3 |
| Israel | 3 |
| Panama | 3 |
| Peru | 3 |
| Turkey | 3 |
| Taiwan | 3 |
| Uganda | 3 |
| UAE | 2 |
| Bangladesh | 2 |
| Colombia | 2 |
| Croatia | 2 |
| Sri Lanka | 2 |
| The Philippines | 2 |
| Romania | 2 |
| Saudi Arabia | 2 |
| Thailand | 2 |
| Zimbabwe | 2 |
| Burkina Faso | 1 |
| Costa Rica | 1 |
| Ecuador | 1 |
| Hong Kong | 1 |
| Iceland | 1 |
| Lebanon | 1 |
| Luxembourg | 1 |
| North Macedonia | 1 |
| Nepal | 1 |
| Sudan | 1 |
| Trinidad and Tobago | 1 |
| Tunisia | 1 |
| Vietnam | 1 |
